# Experimental study on cryotherapy for fungal corneal ulcer

**DOI:** 10.1186/s12886-015-0011-5

**Published:** 2015-03-24

**Authors:** Yingxin Chen, Weijia Yang, Minghong Gao, Michael Wellington Belin, Hai Yu, Jing Yu

**Affiliations:** Department of Ophthalmology, General Hospital of Shenyang Military Area Command, No. 83 Wenhua Road, Shenhe District, Shenyang 110840 China; Dalian Medical University, 9 West Lvshun South Road, Dalian, 116044 China; Department of Ophthalmology, University of Arizona, Arizona Health Sciences Center, 655 N. AlvernonWay, Suite 108, Tucson, AZ 85711 USA

**Keywords:** Fungal corneal keratitis, Cryotherapy, Anti-fungal agents

## Abstract

**Background:**

Fungal corneal ulcer is one of the major causes of visual impairment worldwide. Treatment of fungal corneal ulcer mainly depends on anti-fungal agents. In the current study, we developed an integrated combination therapy of cryotherapy and anti-fungal agents to facilitate effective treatment of fungal corneal ulcer.

**Methods:**

Rabbit models of cornea infection were established using a combined method of intrastromal injection and keratoplasty. After treatment with cryotherapy and anti-fungal agents, scanning electron microscopy, transmission electron microscopy, and confocal microscopy were conducted to observe changes in microstructure in the rabbits. Periodic acid Schiff A and hematoxylin and eosin staining were used for detection of histological changes.

**Results:**

Continuous scanning electron microscopy and transmission electron microscopy observations showed that cryothermal treatment inhibited growth of fungal mycelium by destroying fungal cellular structures. Typical cryotherapy was effective in curing fungal corneal ulcer. Different fungi showed different susceptibilities to treatment. The curative effect of *Candida albicans* was the best, while that of *Aspergillus fumigates *was the worst.

**Conclusions:**

Our study provides a novel method of a combination of cryotherapy and anti-fungal agents for treatment of fungal corneal ulcer. This treatment could help facilitate the practice of fungal keratitis treatment in the future.

**Electronic supplementary material:**

The online version of this article (doi:10.1186/s12886-015-0011-5) contains supplementary material, which is available to authorized users.

## Background

Fungi, especially the filamentous molds, are ubiquitous in the environment and are the major cause of fungal corneal ulcer. Fungal corneal ulcer leads to severe ocular morbidity and blindness worldwide, particularly in developing countries, such as China. Most of the patients with infectious fungal corneal ulcer in China are farmers who tend to ignore eye disorders at the early stage of the disease. Of more concern, those with fungal corneal ulcer do not receive medical care because of discounting the seriousness of the condition, or poverty. A delay in early diagnosis and initiating prompt antifungal medical therapy means that most patients from rural areas in China present with advanced corneal infection by the time they arrive at an eye care facility. As a result, fungal corneal ulcer has become a serious health problem in China [1-3].

Fungal corneal ulcer is usually characterized by severe inflammation, formation of a corneal ulcer, and hypopyon, with the presence of fungal hyphae in the corneal stroma. The majority of pathogens isolated from human cornea with keratomycosis are filamentous fungi, such as *Fusarium* species and *Aspergillus* species [4,5]. There appears to be a strong geographical influence on the occurrence of different types of fungal corneal ulcer, including keratitis due to filamentous fungi and keratitisdue to yeast-like fungi. The proportion of corneal ulcer caused by filamentous fungi has shown a tendency to increase towards tropical latitudes. However, in temperate climates, fungal corneal ulcer appears to be more frequently associated with *Candida* species than with filamentous fungi [5,6].

Therapeutic penetrating keratoplasty and lamellar keratoplasty have been indicated as effective modalities in the treatment of recalcitrant fungal corneal ulcer [7-10]. However, donor corneas are seldom available in developing countries such as China. Moreover, with high rejection and graft failure rates associated with surgery [1,11], other treatment modalities instead of keratoplasty need to be developed.

Cryotherapy combined with anti-fungal agents or a corneoscleral graft has been previously used with success in treating fungal scleritis and keratoscleritis [12,13]. However, the role of this non-keratoplasty alternative in the treatment of fungal corneal ulcer needs to be further investigated. Therefore, in the current study, we developed an integrated therapy combined with cryotherapy and anti-fungal agents for the treatment of fungal corneal ulcer. The effects of treatment were assessed using scanning electron microscopy (SEM), transmission electron microscopy (TEM), confocal microscopy, and histological staining.

## Methods

### Animals

Seventy-seven healthy adult New Zealand White rabbits, weighing 1.5–2.5 kg were used for the establishment of the fungal corneal ulcer model. The rabbits possessed an animal immune certificate with no eye disease. All animal experiments were conducted in accordance with the Institutional Animal Ethics Committee and Animal Care Guidelines of the Department of Ophthalmology, PLA Shenyang General Hospital, Shenyang, which governed the use of experimental animals.

### Preparation of fungal suspension

The fungal strains of *Candida albicans*, *Fusarium solani*, and *Aspergillus fumigates* were provided by the Department of Dermatology, Chinese Medical University. Strains were cultured in Sabouraud mediumat 28°C for 3 days. The spores were collected with 1 mL of physiological saline by washing the surface of the medium. The spore suspension was diluted to a final concentration of 1 × 10^7^ cfu/mL. The concentration was determined under a microscope at 10 × magnification.

### Establishment of the animal model

Previous studies always established fungal corneal ulcer models using the method of intrastromal injection or keratoplasty [14,15]. In the present study, we developed an integrated approach of both methods to improve the rate of success. One week before establishment of the model, the rabbits were injected with dexamethasone sodium phosphate (5 mg/kg) three times in the abdomen. Three days before establishment of the model, the left eye (for experimental use) of each rabbit was administered with levofloxacin (4 mg/mL) four times to clean the conjunctival sac.

Rabbits were anesthetized with 0.05 mL/kg SUMAAN (College of Veterinary Research, The People’s Liberation Army Munitions University) through intramuscular injection. The major components of this anesthetic include dimethyl aniline thiazine and ketamine. The central cornea of the left eye was gently marked with a 6-mm diameter trephine and the corneal epithelium in the drilling groove was scraped with a razor blade. A volume of 0.1 mL of fungal spore suspension of each strain was injected into the corneal stroma. The injured cornea was then covered and sutured with a rabbit corneal graft. The corneal limbus and shallow sclera were stitched up. A volume of 0.1 mL of 1 × 10^7^ cfu/mL fungal spore suspension was injected into the space between the cornea and graft. Ofloxacin (0.3%) was then spread in the conjunctival sac and tarsorrhaphy was performed on the eyes. Forty-eight hours after the operation, corneal grafts were removed and the corneal surface was washed with sterilized physiological saline solution.

Typical fungal corneal ulcers with any positive results from the following examinations were regarded as successful establishment of the model: (1) fungal hyphae were detected by microscopic examination in the corneal tissue; (2) fungi were isolated and cultured from the corneal tissue; and (3) fungal spores or hyphae were detected by confocal microscopy of corneal tissue. Pathological changes in the area of the corneal ulcer, the degree of turbidity, and the anterior chamber reaction were scored according to the published method [16,17] with some modifications. The criteria of pathological scoring are shown in Additional file 1: Table S1. If the model eye scored less than three in all of the indices, the eye was considered to have a small pathological change. If the model eye scored three or more, the eye was considered to have a medium or large pathological change (Additional file 1: Table S2).

### Grouping of the rabbits

Successful establishment of the model was achieved in 53 rabbits. The rabbits were divided into three groups: rabbits injected with *Candida albicans* (*Candida albicans *Group), rabbits injected with *Fusarium solani* (*Fusarium solani* Group), and rabbits injected with *Aspergillus fumigates* (*Aspergillus fumigates* Group). Each group was then randomly assigned into different therapies. In the control group, we administered only medication therapy with fluconazole (2 mg/mL), once per day and natamycin (2.5 mg/mL) twice per day. In the surgery group, we administered cryotherapy and medication therapy with fluconazole (2 mg/mL), once per day and natamycin (2.5 mg/mL) twice per day. Detailed information of the grouping is shown in Additional file 1: Table S2.

### Surgical procedure

Three days after establishment of the model, the rabbits were intramuscularly anaesthetized with 0.05 mL/kg of SUMAAN. The center and edge within 1 mm of the ulcer were carefully removed to expose the stroma of the ulcer and healthy corneal tissue using a sterilized scalpel under a stereo microscope. The corneal lesion was then treated with cryotherapy. Condensation lasted for 7–8 s each time at −50°C using a CO_2_ dry freezer. The corneal edges and conjunctival sac were rinsed with 2 mg/mL of fluconazole and sutured 24 hours after the operation. The stitches were removed and medication was administered as described above.

### Preparation of specimens, and SEM and TEM of the microstructure of pathogenic fungi

The rabbits were sacrificed using an air embolism method. The cornea was removed and fixed using 2.5% neopentyl glycol solution, and preserved at 4°C. For SEM at 3500× magnification, samples before and on the 15th day after treatment were dehydrated, critical point-dried, sticked, coated, and observed with an S-579 scanning electron microscope. For TEM at 8000× magnification, samples before and on the 15th day after treatment were prepared and fixed according to standard methods. Observations were conducted using an H-7500 transmission electron microscope.

### Slit-lamp and confocal microscopic examinations of the effect of cryotherapy on fungal corneal ulcer

For the control group with different fungi, corneal samples that were taken before and on the 30th day after treatment were prepared for slit-lamp observations according to a standard process. For the surgery group with different fungi, corneal samples that were collected before treatment and on the 15th and 30th days after surgery were prepared as described above.

Corneal samples that were taken before and on the 30th day after treatment in the control group with different fungi, and samples that were taken before treatment, and on the 15th and 30th days after surgery were prepared for confocal microscopy as described above.

### Histological examination

Growth patterns of hyphae, and histological changes before and on the 15th day after surgery were assessed using Periodic acid Schiff (PAS) staining. Inflammatory infiltration and histological changes before and on the 15th day after the surgery were observed using hematoxylin and eosin (HE) staining. Observations of the staining were conducted using a microscope at 400× magnification.

### Statistical analysis

The efficiency of treatment was calculated as the rate of cure + significant efficiency rate + improvement rate [18]. The course of the disease was also recorded. The chi-square test was used for abnormally distributed data. All of the analyses were conducted using SPSS version 17.0 (IBM, Armonk, NY) with a significance level of 0.05.

## Results

### Establishment of the rabbit fungal corneal ulcer model

**Figure 1:**
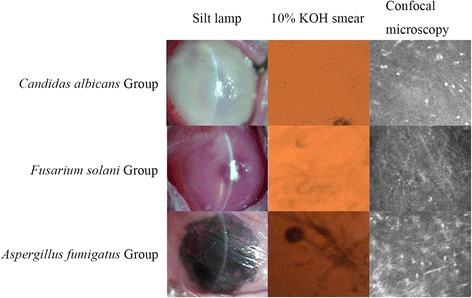
**Fungal keratits caused by fungal infection.** Different panels represent the results of different observations (slit-lamp, 10% KOH smear, and confocal microscopy) of the three pathogenic fungi.

Among the 77 animals, 63 had successful establishment of the fungal corneal ulcer model (82%). However, 10 rabbits were used for pre-experiment and the data for analyses were derived from the remaining 53 rabbits. Different fungal species showed a significant difference in rate of success in establishment of the model. The success rate of the *Candida albicans* group was 91.4%, which was similar to that of 82.4% for the *Fusarium solani* group. However, the success rate of *Aspergillus fumigatus* was 66.7%. The symptoms of corneal ulcer in the different groups are shown in Figure 1.

### Effect of cryotherapy on the microstructure of pathogenic fungal cells

**Figure 2:**
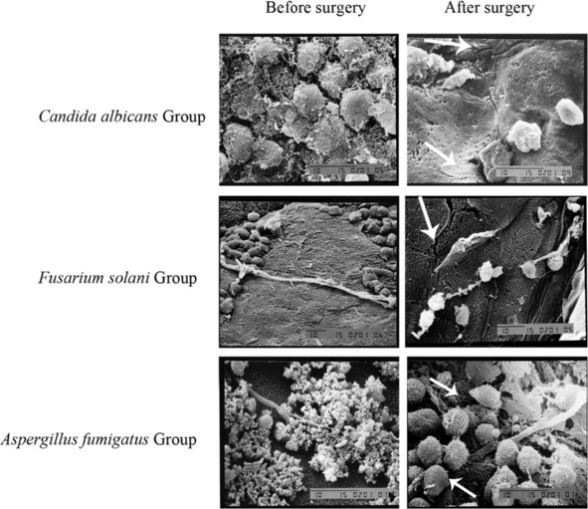
**Fungal structural changes before and after cryotherapy by SEM (3500×).** After treatment, the cells of *Candida albicans* expanded or shrunk, and the cell wall and the cell membrane was fractured. The hyphae of *Fusarium solani *and *Aspergillus fumigates* were diminished or fractured. Cells of the pathogenic fungal crushed, and the cell wall and the cell membrane was fractured. Arrows indicate the detailed changes due to cryotherapy.

The results of SEM showed a large effect of combined therapy on the ultra-structure of fungal species. In *Candida albicans* Group, the cells of *Candida albicans* were expanded or considerably shrunk with the cell wall and the cell membrane fracturing (Figure 2). In Groups *Fusarium solani* and *Aspergillus fumigates*, the hyphae of *Fusarium solani* and *Aspergillus fumigates* were diminished or fractured. Additionally, cells of pathogenic fungi cells crushed with the cell wall and cell membrane fracturing (Figure 2).

**Figure 3:**
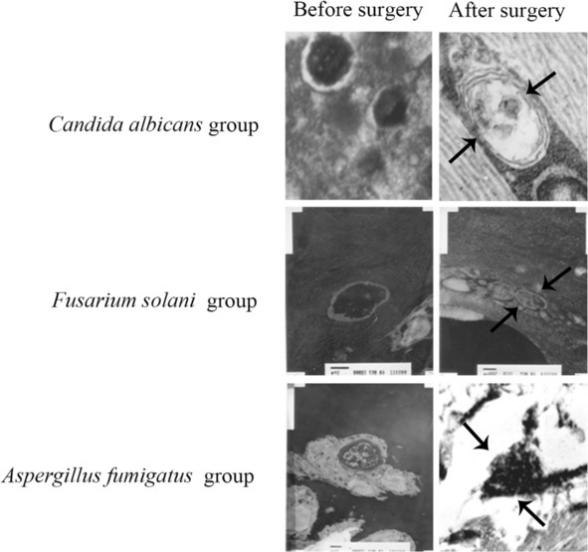
**Fungal structural changes before and after cryotherapy by TEM (8000×).** After surgery, the cells of *Candida albicans* were irregularly spherical, and the cell wall and the cell membrane had disappeared. The integrity of the cells of *Fusarium solani* and *Aspergillus fumigates* was disrupted. Arrows indicate the detailed changes due to cryotherapy.

The results of TEM were consistent with the results of SEM. In *Candida albicans* Group, the cells of *Candida albicans* were irregularly spherical, and cell wall and the cell membrane had disappeared (Figure 3). In Groups *Fusarium solani* and *Aspergillus fumigates*, the cell wall and cell membrane were fractured after surgery and the structural integrity of the cells was disrupted (Figure 3).

### Slit-lamp and confocal microscopic examination of the effect of cryotherapy on fungal corneal ulcer

**Figure 4:**
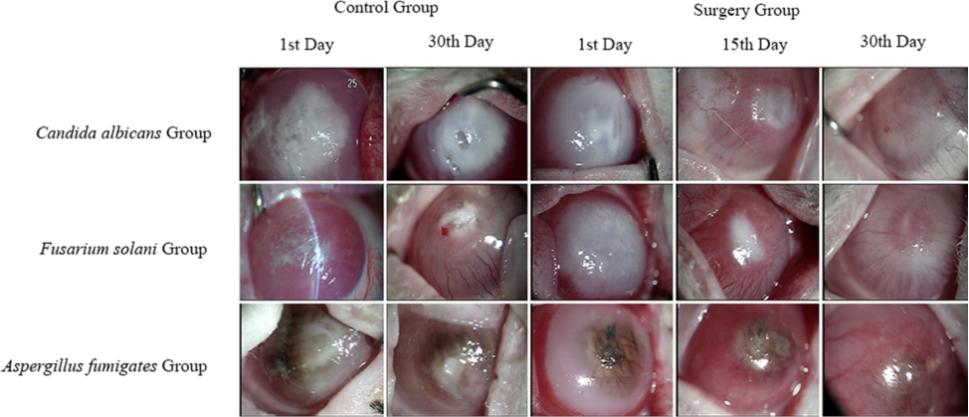
**Effect of cryotherapy on left eye of rabbit fungal corneal ulcer model by slit-lamp microscopy.** In the control group, the structure of corneal tissue was not clear and fungal hyphae were diffusely scattered until the 30th day after treatment. In the surgery group, treatment effect of cryotherapy was observed. Fungal corneal ulcers were healed on the 15th day after surgery.

**Figure 5:**
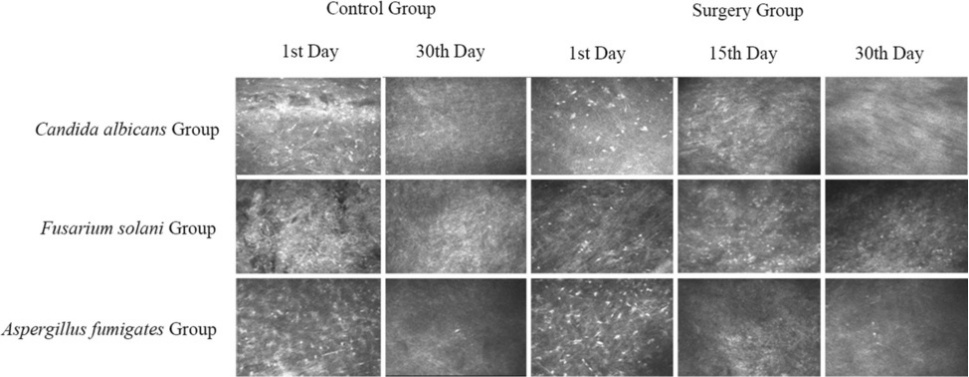
**Effect of cryotherapy on left eye of rabbit fungal corneal ulcer model by confocal microscopy.** On the 1st day in the control group, there was obvious light reflection and diffuse hyphae in the area of the nidus. On the 30th day in the control group, there was a small amount of hyphae in the area of the nidus. On the 1st day in the surgery group, there was obvious light reflection and diffuse mycelia in the area of the nidus. On the 15th day in the surgery group, there was less light reflection and fewer inflammatory cells. On the 30th day, no or a small amount of hyphae were present.

Slit-lamp microscopy showed that the combined therapy had a better treatment outcome for corneal ulcer compared with medication therapy. In the control group with different fungi, lesions did not improve until the 30th day after treatment (Figure 4). However, fungal corneal ulcers were not healed even at the 30th day after treatment, with visible symptoms in the corneas of the rabbits (Figure 4). In the surgery group with different fungi, treatment effect of cryotherapy was observed, with healing of fungal corneal ulcers since the 15th day after surgery (Figure 4). Confocal microscopy showed the same pattern as that with slit-lamp observations (Figure 5).

### Histological examinations

**Figure 6:**
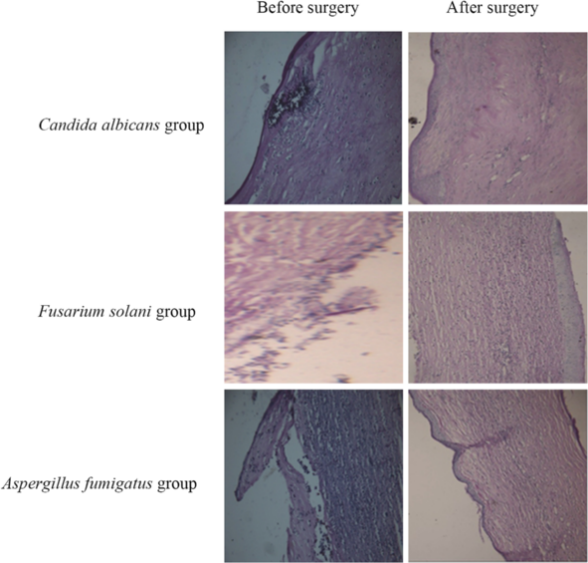
**Histological examination by PAS staining (400×).** After treatment, the squamous epithelium had grown, fibers were neatly arranged, and fungal hyphae were greatly reduced.

**Figure 7:**
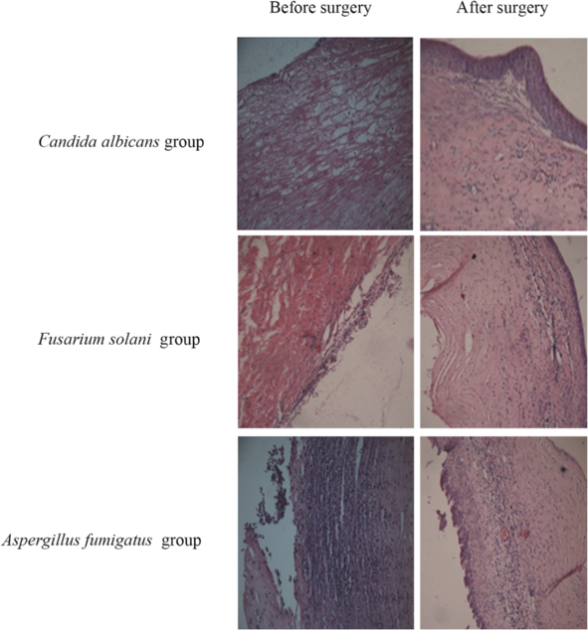
**Histological examination by HE staining (400×).** After treatment, the squamous epithelium had grown, fibers were neatly arranged, and fungal hyphae were greatly reduced.

The results of PAS and HE staining are shown in Figures 6 and 7. A considerable improvement in histological structure was observed for both methods. We observed growth of corneal squamous epithelium, healing of ulcers, and a decrease in the number of inflammatory cells.

### Efficiency of treatment

The overall efficiency of treatment was 74.1% in the surgery group and 34.6% in the control group (Table 1). The disease course of the surgery group (13.75 ± 4.30 days) was significantly shorter than that in the control group (28.66 ± 7.01 days, *P* < 0.05). However, for different pathogenic fungal species, the rate of effective treatment varied. The efficiency of rabbits that were infected with *Candida albicans* and *Fusarium solani* was better than that of rabbits that were infected with *Aspergillus fumigates*, but this difference was not significant (Table 2).

**Table 1:** **Overall treatment efficiency of the surgery and control groups**

	**Successful treatment (n)**	**Unsuccessful treatment (n)**	**Treatment efficiency (%)**
Surgery group	20	7	74.1**
Control group	17	9	34.6

**P < 0.01 compared with the control group.

**Table 2:** **Treatment effect of cryotherapy on different pathogenic fungal species**

**Fungal species**	**Treatment effect**					
	**Surgery group**			**Control group**		
	**Successful treatment (n)**	**Unsuccessful treatment (n)**	**Treatment efficiency (%)**	**Successful treatment (n)**	**Unsuccessful treatment (n)**	**Treatment efficiency (%)**
*Candida albicans*	8	2	80.0**	3	5	37.5
*Aspergillus fumigates*	5	1	83.3**	4	5	44.4
*Fusarium solani*	7	4	63.6**	2	7	22.2

**P < 0.01 compared with the control group.

## Discussion

The incidence of fungal corneal ulcer has been rising in recent years, and the disease is currently the most common infectious corneal disease [11]. In China, the occurrence of fungal corneal ulcer is mainly due to trauma in farmers [19] and has drawn a lot of attention for its potential to lead to blindness. Because of a lack of donor corneas combined with the high cost of transplantation for most people, many serious cases of fungal corneal ulcer result in enucleation of the eyeball. A more economical and effective approach for treating fungal corneal ulcer is imperative. In the present study, we used cryotherapy combined with anti-fungal agents to inhibit excessive tissue damage and improved tissue recovery in fungal corneal ulcer.

For establishment of a fungal corneal ulcer animal model, we selected the internationally accepted corneal injection method combined with the corneal scratch method to improve the rate of success of the model [14,15]. The mean rate of a successful model was 82%, which is consistent with the results of Du et al. (100%) and Liu et al. (83%) [14,16]. However, different fungal species had significantly different success rates. The rate of success of the *Candida albicans* group was the highest (91.4%), and this was similar to the rate of success of the *Fusarium solani* group (82.4%). The rate of success of the model with *Aspergillus fumigatus* was the lowest (66.7%). Our method was applicable for multiple fungal species, in contrast to previous studies, which were all based on single pathogenic fungal species.

In cryosurgery, our study showed that the cooling effect was closely related to the size of the diameter of the freezing point. The best effect was achieved when the freezing point was 1 mm larger than the freezing head, with freezing temperatures ranging from −50°C to − 60°C, and the freezing time ranged from 7 to 8 s. The treatment effect of excision of the ulcer combined with CO_2_ cryotherapy in the present study was promising. The underlying mechanism of this combined therapy may be explained by the following points. 1) Elimination of corneal necrotic tissue could reduce inflammation and the amount of hyphae [20], and improve the effect of cryotherapy and absorption of anti-fungal agents. 2) Rapid cooling could form intracellular ice crystals and disrupt the cell membrane [21]. The re-warming process after surgery could damage the cell membrane again. Moreover, cryotherapy causes denaturation and degradation of proteins within fungal cells [22-25]. 3) Freezing could remove part of the antigen–antibody complexes and reduce the effect of toxins, as well as protein lysozymes [26]. 4) During the re-warming process, the immune system is stimulated. This results in production of interferon by the infected corneal cells, thereby enhancing the body’s resistance to fungi and promoting the growth of healthy tissue [26].

Based on SEM and TEM analyses, we found that the fungal cell wall and the cell membrane were fractured after condensation, and the integrity of the cells was damaged. In addition, the mean disease course was significantly improved in the surgery group compared with the control group. These findings indicated that cryotherapy not only reduced dissolution of the corneal stroma but also significantly shortened the duration of healing.

Moreover, cryotherapy has obvious advantages over conjunctival flap covering surgery or autologous conjunctival grafts. Along with removing pathological tissue, cryotherapy could also reduce infection caused by the central area of corneal opacity and neovascularization. This could lead to better conditions for subsequent penetrating keratoplasty. Cryotherapy also has fewer side effects to surrounding tissues compared with traditional methods. In the current study, the treatment efficiency varied among fungal species, which might be due to the different distribution of hyphae within the nidus. However, this difference was not significant and indicated the treatment potential of the combined therapy against different types of fungal corneal ulcer.

## Conclusions

In conclusion, cryotherapy can dramatically improve the pathological condition and disease course of fungal corneal ulcer with few side effects. This therapy should be taken into consideration in future clinical treatment of fungal corneal ulcer for its effectiveness and non-invasive advantage over traditional methods. Further studies are required to facilitate the application of this method in clinical practice.

## Additional file

Additional file 1: Table S1. Standard of pathological scoring. **Table S2.** Detail pathological conditions of model eyes.
