# Supplementary material for: Experimental study on cryotherapy for fungal corneal ulcer
Source: BMC Ophthalmol. 2015 Mar 24;15:29. doi: 10.1186/s12886-015-0011-5 (PMC4384297; doi:10.1186/s12886-015-0011-5)
Supplement: Additional file 1: Table S1. — Standard of pathological scoring. Table S2. Detail pathological conditions of model eyes. [file 12886_2015_11_MOESM1_ESM.docx]

**Table S1 Standard of pathological scoring.**

| Index | Level | Criterion |
| --- | --- | --- |
| Corneal ulcer area | 1 | 0-50% area of central 6-mm diameter corneal |
|  | 2 | 50-100% area of central 6-mm diameter corneal |
|  | 3 | Area larger than central 6-mm diameter corneal but less than the whole corneal |
|  | 4 | The whole corneal |
| Degree of turbidity | 1 | Slight haze in corneal |
|  | 2 | Grey in superficial corneal and iris texture can be observed through nidus |
|  | 3 | Heavy grey in deep corneal and iris texture cannot be observed |
|  | 4 | Heavy grey in the whole corneal and anterior chamber cannot be observed |
| Anterior chamber reaction | 1 | Cells and cellulose exudates can be observed |
|  | 2 | purulent exudates can be observed |
|  | 3 | Purulent allocated but not connected with nidus |
|  | 4 | Purulent connected with nidus |

**Table S2 Detail pathological conditions of model eyes.**

| Fungal species | Surgery group | |  | Control group | |  |
| --- | --- | --- | --- | --- | --- | --- |
|  | Light pathological changed | Heavy pathological changed | Total | Light pathological changed | Heavy pathological changed | Total |
| *Candida albicans* | 4 | 6 | 10 | 4 | 4 | 8 |
| *Fusarium solani* | 3 | 3 | 6 | 3 | 6 | 9 |
| *Aspergillus fumigatus* | 3 | 8 | 11 | 4 | 5 | 9 |
